# A performance comparison of the fully automated urine particle analyzer UF-5000 with UF-1000i and Gram staining in predicting bacterial growth patterns in women with uncomplicated urinary tract infections

**DOI:** 10.1186/s12894-021-00791-x

**Published:** 2021-02-12

**Authors:** Stephen Shei-Dei Yang, Chun-Chun Yang, Yi-Sheng Chen, Shang-Jen Chang

**Affiliations:** 1 Division of Urology, Department of Surgery, Taipei Tzu Chi Hospital, New Taipei, Taiwan; 2 School of Medicine, Tzu Chi University, Hualien, Taiwan; 3 Division of General Laboratory, Taipei Tzu Chi Hospital, New Taipei, Taiwan

**Keywords:** Urinary tract infection, Diagnosis, Laser flow cytometry, Fully automated urine particle analyzer

## Abstract

**Background:**

The aim of this study was to compare the performance of the new flow cytometer UF-5000 with the UF-1000i and Gram staining for determining bacterial patterns in urine samples.

**Methods:**

Women who attended our clinic with symptoms suggestive of urinary tract infection were enrolled in the study. Mid-stream urine samples were collected for gram staining, urine analysis and urine cultures. Bacterial patterns were classified using the UF-1000i (none, cocci bacteria or rods/mixed growth), the UF-5000 (none, cocci, rods or mixed growth) and Gram staining.

**Results:**

Among the 102 included samples, there were 10 g-positive cocci, 2 g-positive bacilli, 66 g-negative rods, and 24 mixed growth. The sensitivity/specificity of the UF-1000i was 81.8/91.1% for gram-negative rods and 23.5/96.9% for cocci/mixed. The sensitivity/specificity of the UF-5000 was 80.0/88.2% for gram negative rods and 70.0/86.5% for gram-positive cocci.

**Conclusions:**

The UF-5000 demonstrated good sensitivity and specificity for Gram-negative bacilli and demonstrated an improved sensitivity for detecting Gram-positive cocci compared with the UF-1000i.

## Background

Urinary tract infection is a common infectious disease that can cause a significant health care burden, although most cases are uncomplicated infections. It is estimated that 50–60% of women will go through one or more episodes of uncomplicated urinary tract infection (uUTI) in their lifetime [1]. uUTIs usually cause minor symptoms and are rarely life-threatening. However, uUTIs impair quality of life because of their irritative symptoms [2]. uUTIs are diagnosed following the occurrence of positive clinical symptoms and positive urine cultures. The bacterial species that lead to urinary tract infections include *Escherichia coli, Klebsiella* spp., *Enterobacter* spp., *Proteus* spp., *Pseudomonas* spp., *Enterococcus* spp., *Staphylococcus* spp., and *Streptococcus *spp. However, it takes 1–2 days to obtain the results of urine cultures and there is a high contamination rate (0.8–41.6%) during the collection process, which can make interpreting the results difficult or irrelevant [3]. Some experts have suggested that mixed growth in a urine culture may occur among the elderly, the immunocompromised, and those with indwelling catheters, HIV, malignancy, and diabetes [4], but these findings are more commonly regarded as contamination [5]. Manual gram staining of urine specimens has shown a 90% sensitivity when utilized to diagnose bacteriuria, however it is laborious and time consuming [6]. Therefore, a rapid, automatic, reliable and cheaper screening test that can differentiate between gram positive, negative or mixed growth in urine specimens, is required to reduce labor, unnecessary medical costs and waiting time, which would help clinicians improve patient care. Previous studies have examined the efficacy of the UF-1000i (Sysmex UF-1000i; Sysmex Corporation, Kobe, Japan) in differentiating bacilli from cocci/mixed growth [7]. However, the UF-1000i cannot differentiate cocci from mixed growth. Recently, a new model of urine particle analyzer was introduced, the UF-5000 (Sysmex Corporation, Kobe, Japan), where cocci are differentiated from mixed growth bacteria [8, 9]. Therefore, we performed a prospective study to compare the accuracy of gram staining, the UF-1000i and the UF-5000 in differentiating bacterial growth patterns (gram positive, negative or mixed growth) using midstream voided urine specimens from women visiting urological clinics for uUTIs.

## Methods

The current study was approved by the Institutional Review Board at our hospital (IRB No: 05-FS02-024) From July 2016 to June 2019, we prospectively enrolled adult women (aged 20–80 years) who visited our clinic presenting with symptoms suggestive of urinary tract infection. Exclusion criteria were a fever (temperature > 38 °C), urolithiasis, pregnancy, congenital urinary tract anomaly, end stage renal disease receiving dialysis, neurogenic bladder with urethral catheterization, a history of bladder cancer, patients who were immunocompromised and recent antibiotics use (within 7 days). After written informed consent was obtained, the patients were asked to complete a questionnaire, including their baseline characteristics (age, medical history including diabetes or hypertension, childbirth, previous abdominal surgery). They were also asked to complete a urinary tract infection symptom assessment (UTISA) [10], which included 7 symptom categories and 7 quality of life categories, with scores for each assessment ranging from 0 to 3. Patients with a total symptom score of ≥ 4 were regarded as positive for symptoms of urinary tract infection.

At the clinic, the patients were asked to collect midstream voided urine in a sterile container for manual gram staining, routine urinalysis and urine culture. A study nurse instructed the patients on proper collection technique to try and reduce the contamination rate. Only specimens with a bacterial growth of ≥ 10^3^ cfu/mL were included in the comparison of bacterial growth pattern differentiation. A 10 ml sample from the sterile collection cup was poured into a urine sediment centrifuge tube (SY, Shih-Yung medical instruments Co., Ltd, Taipei, Taiwan) for automated urine particle analysis (Sysmex UF-1000i; UF-5000, Sysmex Corporation, Kobe, Japan) within half an hour of receiving the specimen. The gram stains of urine specimens were classified as gram positive, negative or mixed by two experienced clinical laboratory scientists with more than 10 years of experience in the field. For each analyzed sample, the bacteria scatter diagram was classified as rods, cocci/mixed growth or none by the UF-1000i. The bacteria scatter gram was also classified as either gram positive, negative, mixed growth or none by the UF-5000 [9, 11].

### Gram staining

The centrifuged urine from the urine sediment preparation was used to make slides for Gram staining. These slides were air dried, fixed with heat and then stained according to the Gram stain procedure. The slides were then assessed for the presence of bacteria and the staining characteristics were further described. Slides with bacteria were subsequently evaluated for bacterial morphology to determine whether these bacteria were Gram positive or Gram negative. Slides were classified as positive for bacteriuria if ≥ 1 bacteria/HPF was noted. Then, the specimens were classified as gram positive, negative, mixed growth or none [6].

### Microbiological analysis

A 1 µl inoculation loop was applied to the commercial chromogenic agar medium (CPS® ID3, Biomerieux, I′Etoile, France) for the urine culture. The culture plates were then aerobically incubated at 35 °C for 18–24 h. The bacteria were quantified by multiplying the dilution factor by the number of colonies on the agar plate. The growth of more than 2 species of bacteria within the urine culture without a dominant one was regarded as contaminated or mixed growth.

### Statistical analysis

The data within the manuscript are expressed as the mean ± standard deviation. We used MedCalc Statistical Software, version 19.1.3 (MedCalc Software bv, Ostend, Belgium; https://www.medcalc.org; 2019) for the statistical analyses. Nominal or categorical data were compared using a *X*^*2*^ test. Ordinal data were compared using the Mann–Whitney test. Continuous data were compared by an independent t-test. The agreement between two methods was evaluated using kappa statistics. The grading of the agreement complied with Altman’s recommendations (< 0.2: poor agreement, 0.21–0.40: fair agreement, 0.41–0.60: moderate agreement, 0.61–0.80: good agreement, 0.81–1.00: very good agreement) [12]. Prospective sample size calculations were not performed because the agreement between UF-5000 and urine culture was unavailable at the time the pilot study was designed. A p-value of < 0.05 was considered as statistically significant.

## Results

There were 85 patients with no UF-5000 or UF-1000i interpretation and there was 1 specimen with no culture growth, all of which were excluded from the final comparisons. A total of 102 urine specimens from 102 women (mean age = 58.5 ± 18.5 years) with a UTISA score ≥ 4 and bacterial growth ≥ 10^3^ cfu/mL were included for the final analysis. Table 1 summarizes the baseline characteristics of the 102 included patients. The analyzed specimens included 10 g-positive cocci, 2 g-positive bacilli, 66 g-negative rods, and 24 specimens with two or more bacterial species that were regarded as mixed growth (Table 2). Gram-positive bacilli (*Lactobacillus* spp.) were excluded from the agreement analysis. Among the specimens with single bacteria growth, there were Gram positive cocci (2 *Streptococci *spp., 3 *Staphylococci* spp., 1 *Enterococci* spp., *2 Group B Streptococci*, *2 unclassified Gram positive cocci*) and Gram negative rods (53 *Escherichia coli*, 5 *Proteus* spp., 4 *Klebsiella* spp., and 4 *Citrobacter* spp.).

**Table 1:** Baseline characteristics of the included patients

	All n = 102	Menopause(+) n = 56	Menopause(−) n = 44	P value
Age (years, ± SD)	49.56 ± 16.57	62.32 ± 7.55	33.81 ± 9.78	P < 0.001
Diabetes Mellitus, n (%)	12 (11.7)	10 (17.8)	2 (4.5)	P = 0.042
Hypertension, n (%)	21 (20.5)	20 (35.7)	1 (2.2)	P < 0.001
Childbirth, n (%)	65 (63.7)	49 (87.5)	16 (36.6)	P < 0.001
Hysterectomy n(%)	18 (17.6)	17 (30.3)	1 (2.2)	P = 0.001
Abdominal surgery history, n (%)	10 (9.8)	8 (14.2)	2 (4.5)	P = 0.109
Day 0 UTISA score (mean ± SD)	10.71 ± 3.81	10.41 ± 3.73	11.09 ± 4.01	P = 0.383

**Table 2:** The sensitivity of gram stain, UF1000i and UF5000 for specific bacterial species

Bacterial growth of urine specimens	Number (n)	UF1000i	UF5000	Gram stain
*Gram (−)*				
*Escherichia coli*				
≧10^5^ cfu/mL	39	Rods = 38/cocci/mixed = 1	G(−) = 31/G(+) = 3/none = 1/mixed = 4	G(−) = 34/None = 5
10^3^–10^5^ cfu/mL	10	Rods = 3/cocci/mixed = 1/none = 6	G(−) = 6/G(+) = 3/none = 1	G(−) = 4/mixed = 2/None = 4
*Klebsiella* spp.				
≧10^5^ cfu/mL	3	Rods = 3	G(−) = 3	G(−) = 2/G(+) = 1
10^3^–10^5^ cfu/mL	1	None = 1	None = 1	None = 1
*Proteus mirabilis*				
≧10^5^ cfu/mL	2	Rods = 2	G(−) = 2	G(−) = 1/None = 1
10^3^–10^5^ cfu/mL	2	Rods = 1/none = 1	G(−) = 2	G(−) = 1/None = 1
*Citrobacter* spp.				
≧10^5^ cfu/mL	3	Rods = 2/none = 1	G(−) = 3	G(−) = 2/None = 1
10^3^–10^5^ cfu/mL	1	None = 1	G(−) = 1	None = 1
*Gram (+)*				
*Streptococci* spp.	1	cocci/mixed = 1	G(+) = 2	G(+) = 1
*Staphylococci* spp.	3	Rods = 1/cocci/mixed = 1/none = 1	G(+) = 3	G(+) = 2/None = 1
*Enterococci* spp.	1	Rods = 1	G(+) = 1	G(+) = 1
Group B Streptococci	2	None = 2	G(+) = 1/none = 1	G(+) = 1/None = 1
G(+)cocci	2	None = 2	None = 2	None = 2
*Lactobacillus* species	2	cocci/mixed = 1/none = 1	G(+) = 1/none = 1	None = 2
Mixed growth				
≧10^5^ cfu/mL	3	Rods = 1/cocci/mixed = 1	G(+) = 1/G(−) = 1/mixed = 1	Mixed = 2/None = 1
10^3^–10^5^ cfu/mL	19	cocci/mixed = 3/None = 16	G(+) = 5/G(−) = 3/None = 11	G(−) = 1/G(+) = 1/mixed = 2/None = 15

### Gram staining

Among the 102 specimens with both UF-1000i and UF-5000 interpretations, there were only 97 specimens with available gram staining results because timely interpretation by the clinical laboratory scientists was not possible. Of the 97 specimens, 29 were classified as negative. Agreement levels between the results of the gram stain and the urine cultures are listed in Table 3 with a kappa value of 0.48 (moderate, 95% CI 0.36 to 0.60). The sensitivity and specificity of the gram stain for gram negative bacteria were 80.6% and 96.7%, respectively, and the sensitivity and specificity of the gram stain for cocci were 60% and 100%, respectively. The sensitivity and specificity of the gram stain for mixed growth were 18.2% and 97.4%, respectively.

**Table 3:** Agreement levels between the results of gram stain and urine cultures

Urine culture	Gram stain			
	GNB	GPC	Mixed	None
GNB	50	0	2	10
GPC	0	6	0	4
Mixed	1	0	4	16

*GNB* gram negative bacilli, *GPC* gram positive cocci, *Mixed* mixed growth

### UF-1000i

Agreement levels between the results of the Gram-negative bacilli UF-1000i and the urine cultures are listed in Table 4 with a kappa value of 0.49 (moderate, 95% CI: 0.38 to 0.61). The sensitivity and specificity of the UF-1000i for rods were 81.8% and 91.1%, respectively, and the sensitivity and specificity of the UF-1000i for cocci/mixed growth were 23.5% and 96.9%, respectively.

**Table 4:** Agreement levels between the results of UF1000i and urine cultures

Urine culture	UF 1000i		
	Rods	Cocci/Mixed	None
Rods	54	2	10
Cocci/Mixed	3	8	23

#### UF-5000

Agreement levels between the results of the UF-5000 laser flow cytometry and urine cultures are listed in Table 5 with a kappa value of 0.46 (moderate, 95% CI: 0.34 to 0.58). The sensitivity and specificity of the UF-5000 for gram negative bacilli (GNB) were 80.0% and 88.2%, respectively, and the sensitivity and specificity for the gram stain for cocci were 70% and 86.5%, respectively. The sensitivity and specificity of the UF-5000 for mixed growth were 4.5% and 94.9%, respectively. For specific gram positive cocci, the UF-1000i identified all *Staphylococci* spp. (*n* = 3), *Enterococci* spp. (*n* = 1) and *Streptococci* spp. (n = 3), except for one Group B Streptococci and two gram positive cocci (10^3^ and 2 × 10^3^ cfu/mL, respectively).

**Table 5:** Agreement levels between the results of UF5000 and urine cultures

Urine culture	UF 5000			
	GNB	GPC	Mixed	None
GNB	52	6	5	2
GPC	0	7	0	3
Mixed	4	6	1	13

*GNB* gram negative bacilli, *GPC* gram positive cocci, *Mixed* mixed growth

For specific GNB, the UF-5000 identified all *Proteus* spp. and *Citrobacter* spp. except for one *Klebsiella* spp. (3 × 10^3^ cfu/mL). However, the UF-5000 only identified 37 of the 49 *E. coli*.

## Discussion

To the best of our knowledge, this is the first prospective study that compares the efficacy of automated urine flow cytometry systems (UF-1000i, UF-5000), gram staining and urine cultures for urine specimens from women with uUTIs. The results showed that the UF-5000 had good sensitivity (80.0%) for identifying gram negative bacteria with an acceptable specificity (88.2%). With regard to gram positive bacteria, the UF-5000 outperformed the UF-1000i in detecting gram positive cocci (UF-5000 sensitivity: 70% and specificity: 86.5%) with good specificity, which was comparable to gram staining (sensitivity: 60% and specificity: 100%). However, the sensitivity of the UF-5000 for identifying mixed growth bacteria was poor.

The UF-5000 is an automated urine analyzer produced by Sysmex Corporation, which performs flow cytometry analysis with a higher level of accuracy and more precise data [13]. Several previous studies have investigated legacy models of automated urine particle analyzers (including the UF500i and the UF-1000i) for screening urine cultures, while few studies have evaluated the ability of the newer UF-5000 model in the differentiation of bacterial growth patterns. Compared with the legacy systems, the current study showed that the UF-5000 had comparable sensitivity and specificity for GNB (80.0% and 88.2%, respectively). For specific bacteria (Table 2), the current results revealed that the UF-5000 could identify *Klebsiella* spp. *Proteus* spp., and *Citrobacter *spp. but it only identified 37 out of the 49 *E coli*. cases.

However, a retrospective study by Kim et al. [11] reported that the UF-5000 had good performance in identifying *E coli*. Further studies are required to check the performance of the UF-5000 in identifying *E coli*. As for Gram-positive bacteria, the UF-5000 showed high sensitivity and specificity for *Enterococcus *spp., however, the sensitivity for *Streptococci* spp. was much lower. In the current study, the UF-5000 identified all *Staphylococci* spp. (n = 3), *Enterococci* spp. (*n* = 1) and *Streptococci* spp. (n = 3), except for one *Group B Streptococci* (2*104 cfu/mL) and two gram positive cocci (103 and 2 × 103 cfu/mL, respectively). With regard to gram positive bacteria, the UF-5000 outperformed the UF-1000i in detecting gram positive cocci, which was comparable to gram staining.

Gram staining is associated with a sensitivity rate of 88%, a specificity rate of 95%, a negative predictive value of 96%, and a positive predictive value of 84% for identifying bacteriuria [6, 14]. When differentiating bacterial growth patterns, gram staining has good sensitivity and specificity for gram negative (80.6% and 96.7%, respectively) and gram positive bacteria (60% and 100%, respectively). Although real-time reporting of gram staining could reduce the blind initiation of antibiotics, and thus prevent unnecessary expenditure and drug treatment, gram staining is time consuming and labor-intensive. The UF-5000 offers comparable efficacy and a much faster and far easier way of providing the same information, compared with the classic method of Gram staining.

Detecting Gram positive bacteria has significant clinical implications. First, the most commonly isolated Gram-positive uropathogens are *Staphylococcus saprophyticus*, *Enterococcus faecalis*, and *Streptococcus agalactiae*. A previously published review suggested that urologic diseases involving Gram-positive bacteria may be easily overlooked due to the limited culture-based assays that are typically utilized for urine analysis in hospital microbiology laboratories [4]. Hooton et al*.* [12] found that only *Staphylococcus saprophyticus* correlated well with catheterized urine whereas, enterococci and streptococci were poorly correlated with catheterized urine cultures. Therefore, patients with gram positive bacteria shown on the UF-5000 may not have uUTI by classical GNB and may not need empirical antibiotics. Second, in patients with Gram positive cocci as determined by the UF-5000, a urine culture is recommended as well as the administration of antibiotics targeting Gram positive bacteria, as opposed to empirical antibiotics for gram negative bacteria. In this way, patients can avoid unnecessary waiting times, the overuse of antibiotics and increased medical costs. Third, immediately identifying Gram positive bacteria in ascites [13], cerebral spinal fluids [14] and pleural fluids may help clinicians make appropriate and timely antibiotic choices for these life threatening infections. More clinical studies to explore the use of the UF-5000 in these situations is encouraged.

About 21.6% of urine cultures revealed mixed growth (n = 22, 21.6%) and lactobacillus (n = 2, 1.9%), which were regarded as contamination due to improper collection, transportation, preservation or storage. The study only included female participants, which may explain the relatively high contamination rate. Because females have a short on 102 specimens with both UF1000i and UF5000 interpretation, there were only 97 specimens with gram stain results available. There were only 5 specimens that were not examined with gram staining because of unavailability of laborators. Therefore, we did not compare the background differences between the three specimen. urethral length and their urethra meatus is proximal to the vagina and anus, urine specimens from women are more easily contaminated than men. Our study showed that the sensitivity and specificity of the UF-5000 for mixed growth were 4.5% and 94.9%, respectively. Further improvements in laser flowcytometry for the identification of mixed growth could help health care workers and save time, labor and money.

The current study did have some limitations. Although the study was prospective and compared the efficacy of the three methods (gram stain, the UF-1000i and the UF 5000), the major limitation was the limited number of included samples. To prove the efficacy of UF-5000, a sample size of 684 specimens is indicated based on the kappa value (0.46) of the current study (acceptable kappa: 0.6, alpha: 0.05, power: 80%), but the current study only included 102 [15]. In addition, a significant proportion of patient specimens yielded gram negative bacteria and mixed growth culture, so evidence supporting the promising efficacy of the UF-5000 in detecting gram positive bacteria is limited due to the low number of specimens with gram positive bacteria. The menopause can lead to vaginal atrophy and an inability for the vagina to maintain its acidity, which could impact the bacterial strains [16]. Due to the limited number of participants, we did not analyze the effect of menopause on the agreement. A strength of the current study was its prospective nature and the fact that it compares two automated urine particle analyzers. Further studies are still warranted to evaluate the generalizability of the UF-5000 in a larger subset of patients, institutions and populations. In addition, the role of the UF-5000 in detecting bacterial patterns in other type of specimens, *i.e.,* ascites, cerebral spinal fluids and pleural effusion, should be investigated further.

## Conclusions

The UF-5000 demonstrated that is has potential utility as a rapid screening method for bacterial morphology, which correlated well with the legacy analyzer UF-1000i for GNB bacteria, while showing improved sensitivity for detecting Gram-positive cocci.

## Data Availability

The datasets used and/or analyzed during the current study are available from the corresponding author on reasonable request.

## Abbreviations

GNB: Gram negative bacilli
UTISA: Urinary tract infection symptoms assessment score
uUTI: Uncomplicated urinary tract infection
